# Disability, vulnerability and assisted death: commentary on Tuffrey-Wijne, Curfs, Finlay and Hollins

**DOI:** 10.1186/s12910-019-0426-2

**Published:** 2019-11-27

**Authors:** Tim Stainton

**Affiliations:** Canadian Institute for Inclusion and Citizenship, University of British Columbia, 2080 West Mall, Vancouver, BC V6T 1Z3 Canada

## Abstract

This paper builds on the work of Tuffrey-Wijne et al. and explores the issue of vulnerability and persons with disabilities in relation to Euthnasia and Assisted Dying (EAS). The commentary draws on both the literature and on case examples from Canada. Specifically, it considers the issue of EAS as an alternative to, or substituted for, appropriate disability supports. Secondly, it considers the issue of the devaluation of disabled lives in general and within health care practice and ethics. It concludes that current safeguards are inadequate and that as EAS regimes become more permissive the risk to disabled persons will increase.

## Background

Tuffrey-Wijne, Curfs, Finlay and Hollins [1] 2018 paper and a follow up paper [2] present a troubling picture regarding the euthanasia of persons with intellectual disabilities and autism spectrum disorder in the Netherlands. They identify six case reports of people with intellectual disability and three of people with autism in records covering 2012–2016. In a subsequent review covering 2017 and 2018, they identified a further four cases of people with intellectual disabilities, one of whom also had autism spectrum disorder, and three with autism spectrum disorder [2]. In their analysis of the data they raise serious concerns about both the difficulties in assessing whether the patient had made a “voluntary and well-considered request” (one of the legal due care criteria), which as they note, is closely linked to an assessment of the patient’s decision-making capacity, and the stringency of the assessments used to make the above determinations. In their subsequent paper, they raise further concerns about another of the due care criteria which requires that patients are experiencing “unbearable suffering without prospect of improvement”. As they note, ‘This requirement poses a key challenge in relation to euthanasia requests from people with intellectual disabilities and/or autism spectrum disorder, particularly if the nature of suffering for which euthanasia is sought is related to, or affected by, their disability’ [2]. They conclude their 2018 paper with the following reflection: ‘we speculate that many of the challenges highlighted in this paper could also be relevant to patients in the general population, and that they are simply more pronounced or extreme for vulnerable patient groups. It is quite possible that people with intellectual disabilities are like the canary in the coal mine, among the first to come up against issues that turn out to be issues for everyone’ [1].

While Tuffrey-Wijne et al. highlight concerns for persons with intellectual and developmental disabilities, an earlier paper by Kim et al. highlights similar concerns for persons with psychiatric disabilities [3]. They note this is one of the fastest growing populations accessing euthanasia and/or assisted suicide (EAS) in Belgium and the Netherlands, the two jurisdictions currently allowing EAS for psychiatric conditions as the sole underlying cause of suffering. While currently not legal in Canada [4], a recent review of the issue was commissioned by the Government of Canada for consideration when the current law comes up for mandatory review in 2021 [5].

Persons with physical and sensory disabilities can access EAS in both Belgium and the Netherlands with disability as the sole underlying cause of suffering. The Belgian twins, Eddie and Marc Verbessem, are perhaps the most well-known case. Euthanized by lethal injection, the brothers, Deaf since birth and subsequently diagnosed with glaucoma that would lead to blindness, feared dependency and believed being deaf-blind would cause “unbearable suffering.” As Peace notes, ‘Multiple news reports characterized the deaths as a mercy killing. The message was clear: death is a logical and reasonable option if a person will become deaf-blind…. there are some disabilities that are a fate worse than death [6].

Current Canadian law identifies disability explicitly as one of the underlying sources of suffering which would qualify a person to seek EAS, however, the law also requires death to be ‘reasonably foreseeable’ which precludes EAS for persons with disabilities who are not also facing a reasonably foreseeable death [4]. The reasonable foreseeability criteria is however was recently challenged in two cases [7], both of which involved seeking access to EAS for persons with degenerative conditions which create disabilities but whose death was considered not to be reasonably foreseeable. The judgement in the Truchon and Gladu case ruled that the reasonably foreseeable natural death criteria was unconstitutional and therefore invalid. The Lamb case was subsequently withdrawn and the Attorney General did not appeal the Truchon and Gladu case. This opens the door for any persons with a disability to access EAS if they believe their disability causes unbearable suffering.

Concerns regarding the risk that EAS may pose to disabled persons are long standing. The ‘Not Dead Yet’ [8] movement is perhaps the most widely known opponent of EAS by disabled persons. They are however not alone in their concerns. Virtually all of the major disability organizations in Canada have expressed serious concerns regarding the threat euthanasia and assisted suicide pose to the lives of disabled persons [9]. In this commentary, using the literature and case examples from Canada, I would like to explore two core concerns of disabled persons, both signaled in Tuffrey-Wijne et al. While not denying their claim regarding the general population, I will confine my comments to disabled persons generally, though many of the issues will apply more broadly.

First, I will examine the question of EAS as a substitute for, or because of an inability to access, appropriate disability supports. The second issue I want to consider is somewhat more oblique and concerns the way disabled persons lives are devalued within society, health care and ethics. This section, while somewhat more speculative, draws together various strands of evidence to support this proposition. This latter issue clearly affects the first and cuts across the concerns of disabled persons regarding EAS.

### EAS as an alternative to acceptable disability supports

The UN Special Rapporteur on the rights of persons with disabilities, after her visit to Canada, was “extremely concerned” about the implications of assisted dying legislation on people with disabilities after hearing multiple complaints. In her report she states: “I urge the federal government to investigate these complaints and put into place adequate safeguards to ensure that persons with disabilities do not request assistive dying simply because of the absence of community-based alternatives and palliative care [10].”

There have been a number of cases that seem to support these concerns. Archie Rolland died by EAS in 2016. A press report at the time noted: ‘It’s not the illness that’s killing him, Rolland said in a series of emails with the Montreal Gazette. He’s tired of fighting for compassionate care’ [11]. One of the plaintiffs in the Quebec case which challenged the reasonably foreseeable death requirement also indicates it is the nature of the care which is, at least in part, behind his suffering: ‘At a news conference… Mr. Truchon had an assistant read a statement explaining that he couldn‘t face the prospect of life confined to an institution’ [12].

While both these cases seem to indicate the lack of acceptable care options is a major impetus in seeking EAS, a more disturbing case is that of Roger Foley. Foley is a 42 year old man with a degenerative condition. After a lengthy hospital stay, staff are sent, unrequested by Foley, to see if he has an interest in pursuing EAS. Not once, but twice. Foley secretly taped the conversations, which he subsequently released publicly [13]. During one conversation he is informed that if he refuses the home care option available to him he will be charged ‘north of $1500 a day’ if he remains in hospital. Foley indicates that the current option for home care is unacceptable to him as the government-selected home care provider had previously left him in ill health with injuries and food poisoning. When asked if he thinks of harming himself Foley replies he is “always thinking I want to end my life” but quickly clarifies this by saying if he had self-directed support, a service available but not offered to Foley, he would be fine. Foley is currently pursuing legal action regarding his treatment [14].

While the exact nature and motivations for the hospital’s actions are still unclear, what is clear is that Foley was under pressure to leave the hospital without the type of support he considered acceptable or, to consider EAS. It also appears the hospital was suggesting EAS as an alternative to appropriate support. Further, the tapes raise the question of whether EAS was seen as a way to manage the cost the hospital was incurring due to Foley’s refusal to leave.

A final case example involves 41 year old Sean Tagert, a man with ALS, who died by EAS in August of 2019. He was quite explicit that his reason for choosing assisted death was his inability to secure sufficient home care funding in order live a life he considered worth living [15].

While there are many disturbing questions raised by the Tagert and Foley cases, for our purposes the key issue, as with Rolland and Truchon, is the risk EAS poses to disabled persons when disability supports which would allow them to live a life they consider worth living are not readily available. This risk is arguably heighten in the context of austerity and concern with rising health and social care cost. While Foley has chosen to speak out and fight for what he see as his right, one must question how many other people with disabilities like Sean Tagert, out of despair, a lack of knowledge about other options or simply because they are weary of the struggle, will quietly chose to die by EAS.

### Negative valuation of disabled lives

In response to the above, one might be tempted to argue that these are rare exceptions and health care professionals will always seek to prolong life where they believe there is a possibility of a reasonable quality of life. The problem lies in the final part of that statement. The negative perception of the lives of persons with disabilities is well documented as Tuffrey-Wijne et al. note: “Numerous reports in recent years have suggested that the lives of people with an intellectual disability are valued less across society, and that their short life expectancy results from inappropriate value-laden decision-making by healthcare professionals” [1]. Gill in her review of evidence regarding physician attitudes towards disability and the impact on treatment decision found that health professionals tend to hold a negative view regarding the quality of lives of disabled persons and often more negative than that of the general public. She further notes that ‘Research has shown for some time that many health professionals believe life with extensive disabilities is not worth living’ [16]. There is an extensive literature, along with copious anecdotal reports, regarding negative experiences with the health care sector by persons with disability [17]. These range from physical impediments and attitudinal barriers, to reluctance/refusal to provide treatment, refusal of transplants, failure to undertake treatment that would normally be offered to a non-disabled person or undertaking non-medically necessary, highly invasive and high risk interventions [18].

If we look at other intersections of disability and health care, the picture does not improve. Current trends in pre-natal testing indicate a strong negative view towards having a child with a disability [19]. The potential to ‘eliminate Down’s Syndrome’ through pre-natal testing (PNT) and termination is now being discussed widely as a very positive development both with regards to the elimination itself and the potential cost savings which might be realized [20]. Disability scholars have argued that the practice of PNT and termination expresses strongly negative views towards persons with disabilities generally and promotes negative attitudes towards those persons currently living with a disability. Further, it has been argued that these views are the product of a false and biased view about disabled lives as ones of suffering and that suffering is inherent in the impairment itself rather than socially produced [21].

Some scholars have argued that until disabled persons are free of discrimination and receive the supports and services they require to maintain inclusive lives, widespread safeguards are required for PNT [22]. While not directly analogous, EAS is potentially equally influenced by the same negative valuation of disabled lives discussed above and hence it seems reasonable to extend the same caution to safeguards regarding EAS.

A further area which suggest a negative valuation of disabled persons lives in health care is the practice of neo-natal euthanasia. Legal in Belgium and the Netherlands, evidence suggests it is widely practiced elsewhere despite being illegal. A 2005 study [23] found that half the newborn babies who died in Flanders over a recent year-long period (prior to legalization) were helped to die by their physician. Most were premature babies with severe congenital malformations or disabilities and what was described as a poor quality of life, or very premature babies with severe brain damage.

In 2002, the Groningen Protocol for neonatal euthanasia was developed in the Netherlands with the intent to regulate the practice of actively ending the life of newborns and to prevent uncontrolled and unjustified killing. Significant numbers of these cases involve neo-nates with non-life threatening, medically treatable conditions and disabilities, most commonly spina bifida [24]. The American College of Pediatricians note that there is much room for parental, physician, personal, social, and economic bias. In their review of all 22 cases reported to the district attorneys’ offices in the Netherlands from 1998 to 2005 Verhagen & Sauer [24] found that all involved spina bifida. They report that the considerations used to decide on euthanizing were:

- Extremely poor quality of life (suffering) in terms of functional disability, pain, discomfort, poor prognosis, and hopelessness N22 (100%)
- Predicted lack of self-sufficiency N22 (100%)
- Predicted inability to communicate N18 (82%)
- Expected hospital dependency N17 (77%)
- Long life expectancy N13 (59%)

The last consideration, the authors note, is interpreted as the burden of other considerations being greater when the life expectancy is long in a patient who is suffering. What is striking here is that none of these cases were terminal nor apparently experiencing significant physical pain. In all cases, these were largely third party determinations of perceived future quality of life. It is not an unreasonable proposition that similar consideration would influence the practice of EAS.

A further area of concern is the often explicitly negative treatment of persons with disabilities within certain streams of ethics. This is most evident in, but not exclusive to, certain strains of Utilitarian ethics. Singer’s views about disabled persons moral status and the ethics of euthanasia are perhaps best known [25]. He is far from alone however. One of the key concerns from the disability community is the equating of disability directly with suffering. John Harris writes with regards to prenatal testing and elimination of disabled fetuses that ‘where we know that a particular individual will be born ‘deformed’ or ‘disfigured’ …the powerful motive that we have to avoid bringing gratuitous suffering into the world will surely show us that to do so would be wrong’. He goes on to state that in the case of severe disability ‘we should give them a humane death by legalizing euthanasia in such cases’ [26].

One of the key features of all of these arguments is the assumption of ‘suffering’ as inherent to disability. As Tuffrey-Wijne et al. note: ‘the fact that the disability itself, rather than an acquired medical condition, can be accepted as a cause of suffering that justifies euthanasia is deeply worrying [2].’ Where this becomes most concerning is when it is operationalized through approaches like Quality Adjusted Life Years (QALYs) to determine what, if any, interventions offer the best cost benefit outcome [27]. Leading bioethicists [28] have endorsed the view that utilitarianism requires discrimination against the disabled in the allocation of health care resources based on the maximization of quality adjusted life years [29]. As Hilliard states, ‘Consistent with the utilitarian ethic, state sanctioned killing of those deemed to have “lost their dignity” is hailed as a “good” [30]. Studies have shown that nondisabled persons tend to assign lower quality to disabled lives than disabled persons themselves [16, 29]. The outcome then is that disabled persons will virtually always lose in the resource allocation calculus. In the context of EAS, use of QALYs or similar methods raise some very serious concerns. As Barrie notes, ‘problems (with QALY) relate closely to the debate over euthanasia and assisted suicide because negative QALY scores can be taken to mean that patients would be ‘better off dead’ [31].

If the proposition that there is an inherent negative bias towards disability and disabled lives within health care and some ethical norms and systems, it is not difficult to imagine a scenario where disabled persons are counselled, or even encouraged to consider EAS. While speculative at present, there does seem to be enough evidence to urge caution when considering EAS when significant disability is involved.

### Can safeguards fully protect disabled persons?

With regards to the cases reported on in the 2018 article Tuffrey-Winje et al. [1] note:: ‘We found no evidence of safeguards against the influence of the physicians’ own subjective value judgements when considering EAS decision, nor of processes designed to guard against transference of the physicians’ own values and prejudices.’ Eligibility for EAS usually involves and evaluation of ‘suffering’ which as noted above, is often uncritically directly associated with disability. Further, it is generally a bio-medical or psychological assessment and does not take account of the social factors such as the impact of stigma, lack of supports, isolation or institutionalization that may be underlying factors in creating the conditions of suffering. While most jurisdiction are required to consider possible undue influences or pressure that may be motivating the request for EAS, this again tends to be limited to direct pressure, usually from family members. Clearly, it is difficult to account for more diffuse influences which may be coming from inside the health system doing the assessment.

More critically with regard to the issue discussed above regarding EAS as an alternative to acceptable support, current EAS regimes such as the current Canadian legislation, include little or no requirement for meaningful psycho-social assessment of the persons situation and what may be leading to their request for EAS. Additionally little attention is paid to, and there is no requirement to provide, support alternatives that would lessen the suffering which in Tagert’s case would have precluded his accessing EAS. Given the Rolland, Truchon, Foley and Tagert cases discussed above, a comprehensive assessment of their supports and alternatives may well identify options which would mitigate a desire for EAS. Proposals have been put forward in Canada for a system of psycho-social assessment [32] in cases where it is suspected that the suffering may be a result of inadequate or unacceptable social support but have largely been ignored by policy makers and are not part of current legislation or regulation. This type of system would however greatly reduce the risk of disabled persons accessing EAS out of desperation or despair from a lack of supports, which would allow them to live the best life possible with their disability.

## Conclusion

The above should, in my view, raise serious concerns that any safeguards can fully protect disabled persons from an unwanted death as a result of subtle pressure, despair at living in a world where their daily existence is seen as one of inevitable suffering or, exhaustion from fighting for the accommodations required to live a life of dignity and pursue their chosen lifestyle and purposes. As EAS regimes become ever more permissive [33] it is imperative that we step back and look seriously at the issues around disabled and other vulnerable persons. The recent successful legal challenge in Canada to remove the reasonably foreseeable death requirement coupled with calls to allow EAS by advance directive and to extend eligibility to mature minors and those with psychiatric conditions as the sole underlying conditions all present additional concerns for disabled and other vulnerable persons. The reviews by Tuffrey-Wijne et al. [1, 2] coupled with those by Kim et al. [3] and others suggest there are serious risks for vulnerable persons even in current EAS regimes let alone those which are evolving to ever greater permissiveness. It is no longer hyperbole that we are at risk of uncritically heading to a place where the phrase ‘better dead than disabled’ becomes an underlying, if unspoken, driver of policy and practice.

## Data Availability

Not Applicable.

## Abbreviations

EAS: Euthanasia and/or Assisted Suicide
PNT: Pre-Natal Testing
